# Genetic and Environmental Influences on the Allocation of Adolescent Leisure Time Activities

**DOI:** 10.1155/2014/805476

**Published:** 2014-05-20

**Authors:** Brett C. Haberstick, Joanna S. Zeiger, Robin P. Corley

**Affiliations:** Institute for Behavioral Genetics, University of Colorado Boulder, Campus Box 447, Boulder, CO 80309-0447, USA

## Abstract

There is a growing recognition of the importance of the out-of-school activities in which adolescents choose to participate. Youth activities vary widely in terms of specific activities and in time devoted to them but can generally be grouped by the type and total duration spent per type. We collected leisure time information using a 17-item leisure time questionnaire in a large sample of same- and opposite-sex adolescent twin pairs (*N* = 2847). Using both univariate and multivariate genetic models, we sought to determine the type and magnitude of genetic and environmental influences on the allocation of time toward different leisure times. Results indicated that both genetic and shared and nonshared environmental influences were important contributors to individual differences in physical, social, intellectual, family, and passive activities such as watching television. The magnitude of these influences differed between males and females. Environmental influences were the primary factors contributing to the covariation of different leisure time activities. Our results suggest the importance of heritable influences on the allocation of leisure time activity by adolescents and highlight the importance of environmental experiences in these choices.

## 1. Introduction

Adolescents are confronted with a large number of choices regarding their use of free or leisure time. Of the many options, they could choose to participate in extracurricular activities such as competitive sports teams or relatively unstructured activities such as socializing with their peers or solitary, passive, or sedentary activities such as watching television. Some adolescents may allocate a large amount of time to family, caring for younger members or doing housework, while others work in paid employment situations outside the home. How adolescents allocate their time is of importance as participation in social, physical, and passive types of activities has been associated with the quality of academic performance, physical and psychological health, and behavioral problems both concurrently and at subsequent ages [1–4].

Much of the research into how adolescents spend their time outside of school and work has focused on leisure time activities. So, appropriately, recent estimates from the American Time Use Survey (ATUS) suggest that both male and female adolescents (ages 15–19 years) in the United States spend more than five hours a day on leisure time activities, making leisure time second only to personal care activities (e.g., sleeping, bathing, and dressing) as a proportion of daily life, exceeding time spent at both school and work [5]. However, the definition of what constitutes leisure time varies between studies with regard to the classification of these activities. Although there is an absence of a standard definition in the literature, leisure time is operationalized as being either the amount of time spent engaged in activities or type of activity, but it could also be classified by its psychological and emotional impact [6]. Further, all aspects of leisure time will differ as a function of gender, race, age, and cultural influences [4, 7, 8].

Leisure time activities have been assessed in a number of ways, but self-report questionnaires remain the most widely used method for assessing a wide variety of leisure time activities [9]. In contrast to technology-based tools (e.g., accelerometry), self-report questionnaires provide a low cost and easy to use method for encompassing the diversity of adolescent out-of-school activities. Response bias is one important limitation, however, and may be due to pressures to respond in a socially acceptable manner. Both physical activity and sedentary behaviors have been shown to be sensitive to this type of bias [10, 11], though not always [12].

Family, economic circumstances, and peers have all been implicated as meaningful influences on the allocation of leisure time [1, 13–15]. However, heritable influences may also be an important influence. Twin studies are well suited for investigating the extent to which genetic and environmental influences contribute to individual differences in how siblings of the same family allocate leisure time. Heritable influences are implicated when the monozygotic (MZ) twin correlation is twice the dizygotic (DZ) twin correlation, as MZ twins share all of their genes in common while DZ twins share only half or 50%. Shared environmental influences are those experiences that make siblings of the same family similar and are suggested when the DZ twin correlation is greater than half the MZ twin correlation. In situations where the DZ twin correlation is less than half the MZ twin correlation, nonadditive genetic or dominance contributions to individual differences are suggested.

Twin and family studies have primarily focused on leisure time physical activity and passive activities such as watching television. In general, the magnitude of genetic influences on individual differences ranges between zero and 85% with differences as a function of age, sex, the duration of physical activity, and means of assessment [16–23]. Shared environmental factors appear to have important influences on physical activity during childhood and early adolescence, with their effects diminishing into late adolescence and young adulthood [16]. To our knowledge, only a few twin studies of passive leisure time activities [24–26] have been reported and generally implicate increasing heritable influences as children age and decreasing contributions from environments that make siblings of the same family more alike.

Although physical and passive activities are both widely engaged in by adolescents, they are only two of a variety of activities in which adolescents participate. Other important activities include social, family, and intellectual pursuits. Along these lines, our analyses were designed to examine two questions. First, to what extent do genetic and environmental influences contribute to individual differences in adolescent leisure time activities? Second, to what extent do the genetic and environmental influences on one leisure time activity also influence other activities?

## 2. Methods

### 2.1. Subjects

A total of 2847 adolescent twins from 1429 families (11 families with only 1 twin) were drawn from the Longitudinal Twin Study [27] and the Community Twin Study [28, 29]. Zygosity status was determined by a combination of tester ratings and DNA polymorphisms, with discrepancies between testers and DNA results resolved by a second round of genotyping. Nine hundred twenty-four DZ same-sex twins (50.2% female), 1374 MZ twins (54.9% female), 547 DZ opposite-sex twins (50.3% female), and one pair of males whose zygosity was ambiguous due to DNA refusal completed questionnaires at an average age of 15.1 years (S.D. 2.2 years). The percentages of 12- to 18-year-olds in the total sample were roughly comparable (ranging from 10.0% to 22.9%), with higher proportions of 12- and 14-year-olds. The sample was self-identified as 86.5% White, with 7.8% endorsing multiple racial origins. Approximately 10% of the sample was identified ethnically as Hispanic.

### 2.2. Measures

Leisure time activity was measured with 17 items drawn from a questionnaire expanded from [30]. Leisure time activities were rated on a six-point Likert scale ranging from no hours spent (0), one hour or less (1), two to three hours (2), four to five hours (4), six to seven hours (5), or eight or more hours (6) spent on an activity after school or work and on weekends. Two items about the amount of time (hours) per week spent watching television on weekdays and weekends were summed into a single variable. A total leisure time composite representing the sum of all 17 items was created for comparison purposes after transformation of the Likert items into hour equivalents.

### 2.3. Analyses

Age trends within sex for each item were examined by regression serially on linear, quadratic, and cubic age terms. To determine which items formed coherent scales, unstandardized residuals after taking out significant age effects were then subjected to an exploratory factor analysis (principal components, varimax rotation) using SPSS, version 22. Based on these results, five leisure time scales were created and included: physical, social, intellectual, family, and passive activities. These five scales represent sums of age-corrected residual items.

Twin correlations and genetic models were estimated with sex as a covariate in the statistical software Mx [31]. Two genetic models were utilized for the current analyses: sex limitation and Cholesky decomposition [31, 32]. When based on data from same-sex sibling pairs, the sex limitation model examines whether the magnitude of heritable and environmental contributions to leisure time allocation differs between males and females (quantitative sex differences). When data are also available from opposite-sex sibling pairs, additional sex-specific parameters can be included in order to examine whether different factors contribute to leisure time activities in one sex but not the other (qualitative sex differences).

**Figure 1:**
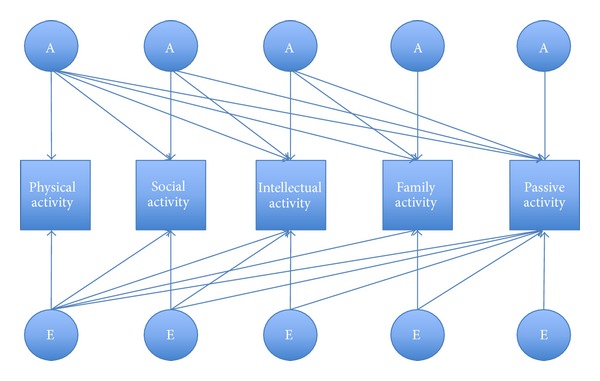
Cholesky decomposition model. This model decomposes the covariance between different leisure time activity domains into that due to genetic (G), including additive (A) genetic, and environmental, including shared (C) and nonshared (E), influences.

Although useful, univariate twin models may not provide enough statistical power to choose between genetic and environmental influences on a particular leisure time activity domain and may provide an overestimation of the heritability. When additional variables have been measured from the same individual, multivariate models can be more statistically powerful as they make use of all the covariance with other leisure time activity domains. Therefore, we also fit a Cholesky decomposition model to our data. This model examines the extent that genetic and environmental influences contribute to the covariation of different leisure time activities and is a simple restatement of that latent factor structure designated in our univariate models. Latent genetic (A) and environmental (C and E) influences are stratified into influences that are common to leisure time activities and those that are specific or residual to one activity (Figure 1). From this model, it is possible to obtain the genetic (environmental) correlation, which indexes the extent that genetic (environmental) influences are common to different leisure time activity domains.

The fit of our genetic models was evaluated using maximum-likelihood estimation. Our baseline model included the additive genetic (A) and nonshared environmental (E) latent factors and either a nonadditive genetic (D) or shared environment (C) factor, as both are confounded in sibling-based models. The significance of model parameters was evaluated by a comparison of twice log-likelihood (−2LL) for models with or without the parameters, with the difference distributed as a chi-square distribution and the degrees of freedom being equal to the difference between the number of parameters estimated. A nonsignificant difference in chi-square (Δ*χ* ^2^) between two models indicates that the parameters dropped from the more parsimonious model were not significantly different from zero. Models were accepted on the basis of the Akaike information criterion {AIC) [33] as calculated by subtracting twice the difference in the degrees of freedom from the difference chi-square between any particular model and the fullest, that is, least parsimonious, model considered. The AIC indexes the extent that a given model offers the most parsimonious, but adequate, explanation to the data, though limitations to using the AIC as a primary criterion in evaluating model fit do exist [34].

## 3. Results

For each of the 17 leisure time activities, the percentage of adolescents who spent no time per week doing an activity, eight or more hours per week doing an activity, and the mean number of hours per week is reported in Table 1. Of the 17 activities, three were not engaged in by over half of the sample, with the least frequently reported activity being taking care of younger family members (76%). Only three percent of the sample reported not watching television during an ordinary week. Conversely, one-third of the sample reported viewing television for more than eight hours per week. Spending time with friends, doing schoolwork, and taking part in organized sport were the activities that the highest proportions of adolescents in this sample spent eight or more hours doing per week, and it was to an extent similar for males and females. For most items, residuals after correcting for age trends within sex are highly correlated with the uncorrected hours per week (Table 1). The exceptions are for the two friends items and talking on the telephone where hours spent per week increased significantly with age in both sexes.

Summing across items yielded a total leisure score with means of 44.1 (standard deviation, S.D. = 16.7) hours for males and 43.5 (S.D. = 17.0) hours for females, or approximately six hours per day. Scores on a total leisure time scale ranged between 2 and 120 hours, with a slight upward skewness. The 1.2% of the sample who reported spending total leisure times of 90 hours or more per week was elevated on every item but had ranges comparable to the sample as a whole.

Principal component analyses yielded five leisure time scales with eigenvalues above one and factor loading ≥0.40. These included physical, social, intellectual, family, and passive activities. Factor loadings for each of the 17 leisure time activities are provided in Table 2. All but two items, doing things with family and sitting and listening to music, could be clearly allocated to a particular scale. These two items were subsequently allocated to the family and passive scales, respectively, in order to preserve a simple structure in the scales and to create scales that reflected clear domains of leisure activities. Cronbach alphas ranged from 0.36 for the passive scale to 0.69 for the physical activity scale.

Phenotypic correlations for males and females for the five leisure time scales are shown in Table 3. Generally, the phenotypic correlations between the five leisure time scales were positive and small (0.08) to moderate (0.35) in magnitude for both sexes. Negative small phenotypic correlations (from −0.03 to −0.01) were observed between the physical activity, passive, and intellectual scales for both males and females. This suggested the possibility of different etiological influences on the hours allocated to these three types of leisure time activities.

Twin correlations for each leisure time scale are shown in Table 4. Overall, MZ male and female twins were more similar than same-sex and opposite-sex DZ twins. This pattern of correlations suggests genetic influences on the amount of time spent engaging in different types of leisure time activities. The greater than half MZ twin correlation for physical activity and passive leisure time activities for male DZ twins suggests that environmental influences shared by siblings of the same family are important sources of individual differences. The lower opposite-sex than same-sex DZ twin correlations suggest the possibility of different latent influences for males and females.

### 3.1. Univariate Genetic Modeling

Table 5 summarizes the results from our baseline (full) and best-fitting univariate models. The baseline model allowed A, C, and E latent influences to be estimated separately between males and females (quantitative sex differences). Sex-limited genetic influences were also included in our baseline model to test whether the same genes contribute to leisure time activities between males and females (qualitative sex differences). The model fit for each of the baseline models of five leisure time activity scales is provided in Table 5.

Against the baseline model, we next compared the fit of models that equated the latent A, C, and E parameters for males and females. For each of the scales, genetic and environmental influences could not be equated between the sexes without a significant deterioration in model fit (*P* ≥ 0.01). Results from models that equated the DZ and OSDZ twin genetic correlations to be equal indicated that the same genes were influencing leisure time activities in both sexes (*P* ≤ 0.24). Similar results were obtained from models that equated shared environment correlations between DZ and OSDZ twins (*P* ≤ 0.20). These findings indicated that although the allocation of leisure time activity was influenced by the same genes in males and females, the magnitude of their impact was different between the sexes.

To refine our baseline model further, we next compared the fit of a series of nested submodels that dropped either A, C, or both latent factors in males and females separately. For males, C influences were found to be important contributors to the hours spent engaged in physical (Δ*χ* ^2^ = 15.95, Δdf = 1, *P* > 0.001, and AIC = 13.95) and passive leisure time activities (Δ*χ* ^2^ = 4.885, Δdf = 1, *P* > 0.03, and AIC = 2.88). The best-fitting models for the social, intellectual, and family leisure time scales included only A and E influences. For females, C influences were important influences on intellectual (Δ*χ* ^2^ = 5.53, Δdf = 1, *P* = 0.02, and AIC = 3.53) and passive (Δ*χ* ^2^ = 4.88, Δdf = 1, *P* = 0.027, and AIC = 2.88) leisure time activities but not for physical (Δ*χ* ^2^ = 0.74, Δdf = 1, *P* = 0.39, and AIC = −1.27), family (Δ*χ* ^2^ = 2.74, Δdf = 1, *P* = 0.10, and AIC = 0.74), and social (Δ*χ* ^2^ = 2.43, Δdf = 1, *P* = 0.12, and AIC = 0.44) leisure time activities, where C influences could be dropped from the model for females without a significant deterioration in model fit. The best-fitting model and variance component estimates for each of the five activity scales are shown in Table 5.

### 3.2. Multivariate Genetic Modeling

We next investigated the extent of familial specificity in the genetic and environmental influences on physical, social, intellectual, family, and passive leisure time activities. Because our univariate models indicated that shared environmental influences were important sources of individual differences, we included A, C, and E latent factors in a five-variable Cholesky decomposition model. Latent factors were allowed to differ between the sexes.

The overall fit of our Cholesky model was −2LL = 87184.10, df = 14075. Parameter estimates for the genetic and environmental contributions to individual differences in five leisure time activities and the covariance between the different activities are shown in Table 6 for males and Table 7 for females. Bolded estimates indicate that a parameter is statistically significant as judged by its 95% confidence interval (95% CI). As shown, the magnitude of heritable and environmental effects varied for each of the five leisure time activities (along the diagonal), with similar magnitudes to those obtained from our baseline or full univariate models. For males, the covariation of different leisure time activities appears to be largely due to environmental influences, as only family leisure time activities evidenced the influence of genetic factors that also contributed to intellectual activities. For females, genetic contributions to physical activity were also found to influence intellectual leisure time activities. Nonshared environmental influences were the largest contributors to the covariation of different leisure time activities.

In order to understand the proportion of variance different leisure time activities shared, we estimated the genetic and environmental correlations that are presented in Tables 8 and 9 for males and females, respectively. Bolded estimates indicate that a parameter is statistically significant as judged by its 95% confidence interval (95% CI). As shown, few leisure time activities shared common genetic influences for either sex. For males, similar genetic influences appear to be contributing to both intellectual and family leisure time activities (*r*_*g*_ = 0.41, 95% CI: −0.04, 0.1.0) whereas for females common genes contributed to both social and intellectual activities (*r*_*g*_ = 0.51, 95% CI: −0.49, 0.1.0). None of the five leisure time activities were influenced by common shared environmental contributions. For both males and females, however, nonshared environmental experiences on different leisure time activities were often common to each other. The exception to this is the nonsignificant nonshared environmental correlation between physical leisure time activity and passive activities.

## 4. Discussion

Leisure time activities are an important part of many adolescents' days and are often the predominate choice the majority of children make. Because leisure time activities have been linked with healthy and unhealthy lifestyles as well as educational attainment [1–4], we sought to determine etiology of individual differences in the allocation of leisure time activities. To do so, we examined self-reported hours spent engaged in five leisure time activity domains among adolescent same- and opposite-sex twin pairs. Using a twin design to understand the etiology of individual differences in the allocation of leisure time allowed us to investigate three questions. First, to what extent do genes and environments contribute to individual differences in the allocation of leisure time? Second, are there sex differences in the heritable and environmental influences on the time spent engaged in leisure time activities? Lastly, to what extent are genetic and environmental influences specific to a particular activity domain or shared across different domains?

Our first two questions sought to determine the type and magnitude of genetic and environmental influences on the allocation of leisure time and whether they differed between sexes. Existing twin studies have implicated genetic influences on exercise and sport participation and general physical activity. The estimates of the size of genetic effects vary between studies, ranging from zero to 85% [16–23, 35]. Sex differences in the magnitude of genetic contributions have also been implicated, with greater effects among males than females, especially during adolescence [35–37]. Shared environmental contributions to physical activity levels have also been suggested in adolescent samples [16, 23, 24, 38] with estimates ranging between 25% and 75%. Against this literature, results from our study are broadly consistent with findings that implicate genetic and shared environmental influences on adolescent physical activity, though they differ in respect to the magnitude of genetic effects. One possible reason for this could be that twin siblings played the same or different sports. Though they had different amounts of practice or playing time, they live in a household where there is an emphasis placed on sports participation. Though it was not possible to determine if this was the case in our data, a scenario such as this would be expected to result in DZ twin correlations greater than half the MZ twin correlation. A further possibility could be changes in the role genetic and environmental influences have during adolescence [35].

Declining physical activity during adolescence [39–41] has sparked a growing interest in the etiological influences on sedentary or passive leisure time activities. The increase in the hours spent engaged in passive leisure time activities has been linked to poor metabolic syndrome profiles [42–44] as well as poorer psychosocial functioning [45–47]. Further, previous work has suggested that physical and passive activities are not two ends of a spectrum of activity and that the two types of behaviors are relatively distinct [48–50]. To date, there have been few heritability studies of sedentary behaviors. In general, they implicate heritable and environmental influences with estimates that vary widely between males and females [24–26]. Consistent with this previous literature, we found both genetic and shared environmental contributions to the hours spent engaged in passive leisure time activities and that their magnitudes differed between the sexes. Differences between males and females could reflect family expectations.

Because adolescents have wider choices in how to spend their leisure time, we also examined the genetic and environmental influences on additional leisure time activities. These included social, intellectual, and family activities that are thought to provide important opportunities for adolescents to develop skills and interests and promote growth [2, 51–53]. To our knowledge, the etiology of individual differences in these three leisure time activities has not been examined previously. From both our univariate and multivariate analyses, we found that each activity domain evidenced small to moderate genetic influences and large nonshared environmental effects, which may reflect measurement error. Difference in the parameter estimates may be due to the lower statistical power from considering each measured activity domain separately as in the case of our univariate models. Shared environments, such as home and school experiences, were also important influences on intellectual activities for females but not males. That intellectual leisure time activities evidence the impact of environmental experiences shared by siblings of the same family suggests that educational programs and opportunities as well as the parental prioritization of learning that are directed towards females would be expected to offer an opportunity or target for intervention efforts to improve or further enhance these activities.

Our third question sought to investigate the extent that genetic and environmental influences on one activity domain also influenced other activity domains. A common observation from our Cholesky model was that environmental influences, both shared and nonshared, were the primary etiological influences in the covariation of different leisure time activities. In the case of physical activity and social activities among males, an example of a shared or common environmental experience could be a team's emphasis on social interactions amongst its members and the large amounts of time they spend together in an athletic environment. Similarly, the relationship between social and passive activities among males could reflect an adolescent choice of activity while spending time with peers. Further, results from our multivariate models indicated that genetic influences on leisure time activities were largely specific to that activity. Further, results from our Cholesky model indicated that genetic influences on leisure time activities were largely specific to that activity. Among males, our results suggest that the time allocation to intellectual and family leisure time activities has similar genetic influences. Among females, a similar relationship was identified for physical activity and social leisure time activities. This could reflect genetically influenced interaction styles or personality characteristics that are involved in these activities. Future studies, though, are needed to examine these possibilities.

Although our results are largely consistent with the extant literature, a number of limitations need to be considered when generalizing these results. First, leisure time activities were assessed via self-report which may be impacted by recall bias. Similarly, social desirability or a tendency to report more socially favorable responses may have resulted in an overreporting of the time allocated to the different activities [9]. Second, though the passive leisure time activity scale assessed watching television, it did not include other measures of computer and video game usage that are common activities of many adolescents. Third, though we controlled age and sex effects on leisure time activities, we did not control possible differences in sociodemographic influences. Lastly, our measure of leisure time activities may reflect those engaged in by American youth and thereby limit their generalizability.

In conclusion, we sought to determine the type and magnitude of heritable and environmental influences on adolescent leisure time allocations and the extent to which the genetic and environmental influences on one leisure time activity domain also influenced other activity domains. To this end, our findings suggest that both genes and environments, especially those environmental experiences shared by siblings of the same home, are important contributors to how leisure time is allocated. Importantly, though the magnitudes of genetic and environmental influences appear to differ between males and females, having an awareness of the extent that leisure time allocations are environmentally influenced may offer opportunities for effective public health messaging and interventions designed to change unhealthy behaviors as well as to promote certain types of activities. That environmental influences also contribute to the covariation of leisure time activities suggests the possibility of influencing more than a single choice of how children allocate their leisure time.

## Figures and Tables

**Table 1:** Descriptive statistics for 17 leisure time activity items.

Leisure time items	0 hours (%)	8+ hours (%)	Males	Females	*R* ^2^
			Mean (S.D.)	Mean (S.D.)	
Taking part in an organized sport or recreation program?	29	17	3.69 (3.12)	3.23 (3.05)	1.00
Working out as part of a personal exercise program	39	5	2.25 (2.50)	1.87 (2.22)	0.99
Playing pickup games like basketball, touch football, and so forth?	44	4	2.38 (2.45)	1.44 (2.15)	0.97
Practicing different physical activities?	39	6	2.33 (2.56)	1.96 (2.42)	0.99
Going out with friends or dating?	25	13	3.23 (2.87)	3.37 (2.85)	0.89
Sitting around with friends?	12	19	4.36 (2.83)	4.03 (2.81)	0.95
Talking on the telephone?	33	6	1.69 (2.09)	2.58 (2.56)	0.96
Doing your schoolwork?	12	16	3.85 (2.71)	4.43 (2.77)	0.98
Reading for fun?	48	3	1.18 (1.82)	1.79 (2.26)	0.98
Doing things with a club?	70	3	0.89 (1.85)	1.01 (1.94)	0.99
Spending time on a hobby?	38	5	2.25 (2.52)	1.90 (2.27)	1.00
Doing things with your family?	15	7	3.21 (2.31)	3.25 (2.39)	0.99
Taking care of younger family members?	76	2	0.62 (1.48)	0.92 (1.95)	1.00
Doing household chores?	27	2	1.92 (1.80)	2.01 (1.86)	1.00
Total hours watching television weekday plus weekend	3	30	6.74 (4.65)	6.17 (4.53)	0.99
Just sitting around doing nothing?	50	2	1.44 (1.93)	1.23 (1.88)	0.99
Just sitting and listening to music?	32	5	2.07 (2.26)	2.27 (2.40)	0.98

Note: S.D.: standard deviation; *R* ^2^: correlation between the raw data and residual data.

**Table 2:** Factor loadings of residualized leisure time activity hours on principal components.

Leisure time items	PC1	PC2	PC3	PC4	PC5
Taking part in an organized sport or recreation program?	**0.72**	−0.05	0.23	−0.13	−0.10
Working out as part of a personal exercise program	**0.54**	0.26	0.08	0.10	−0.14
Playing pickup games like basketball, touch football, and so forth?	**0.73**	0.13	−0.04	0.16	0.07
Practicing different physical activities?	**0.76**	0.09	0.20	0.08	0.05
Going out with friends or dating?	0.16	**0.80**	0.04	−0.02	−0.06
Sitting around with friends?	0.08	**0.76**	0.12	−0.10	−0.02
Talking on the telephone?	0.12	**0.64**	−0.08	0.12	0.14
Doing your schoolwork?	0.15	0.01	**0.48**	−0.11	−0.22
Reading for fun?	−0.18	−0.01	**0.58**	0.30	−0.08
Doing things with a club?	0.17	−0.04	**0.59**	−0.08	−0.01
Spending time on a hobby?	0.15	0.19	**0.58**	0.13	0.17
Doing things with your family?	0.25	0.02	0.40	**0.38**	0.11
Taking care of younger family members?	0.02	−0.04	−0.07	**0.77**	−0.06
Doing household chores?	0.12	0.11	0.14	**0.72**	0.08
Total hours watching television weekday plus weekend	0.07	−0.01	−0.09	−0.02	**0.74**
Just sitting around doing nothing?	−0.11	0.08	0.02	0.01	**0.73**
Just sitting and listening to music?	−0.07	0.48	0.06	0.20	**0.42**

Note: PC: principal component.

**Table 3:** Phenotypic correlations between five leisure time activities for males (below the diagonal) and females (above the diagonal).

Leisure time scale	Physical activity	Social activity	Intellectual activity	Family activity	Passive activity
Physical activity	—	0.23	0.35	0.20	**−0.03**
Social	0.29	—	0.08	0.11	0.22
Intellectual	0.26	0.17	—	0.26	**−**0.07
Family	0.28	0.14	0.29	—	0.10
Passive	**−0.01**	0.17	**−0.02**	0.11	—

Note: all phenotypic correlations are significant at *P* < 0.01, except bolded cells, which indicate a nonsignificant correlation.

**Table 4:** Twin correlations (95% confidence intervals) for MZ and DZ same- and opposite-sex twin pairs.

Zygosity	Leisure time activity scales				
	Physical activity	Social activity	Intellectual activity	Family activity	Passive activity
MZM	0.51 (.42, .59)	0.50 (.41, .57)	0.41 (.31, .49)	0.50 (.41, .57)	0.21 (.41, .32)
MZF	0.52 (.42, .59)	0.54 (.46, .61)	0.39 (.30, .47)	0.54 (.46, .61)	0.54 (.47, .61)
DZM	0.42 (.31, .52)	0.21 (.09, .32)	0.19 (.06, .32)	0.21 (.09, .32)	0.24 (.09, .32)
DZF	0.28 (.15, .39)	0.30 (.18, .41)	0.23 (.11, .34)	0.30 (.18, .41)	0.30 (.18, .41)
OSDZ-MF	0.07 (−.09, .23)	0.27 (.12, .41)	0.09 (−.05, .23)	0.19 (.03, .33)	0.13 (−.02, .28)
OSDZ-FM	0.12 (−.07, .29)	0.42 (.26, .54)	0.03 (−.15, .21)	0.06 (−.13, .23)	0.23 (.05, .39)

Note: MZ: monozygotic; DZ: dizygotic; OS: opposite-sex; M: male; F: female.

**Table 5:** Model fit statistics and variance component estimates (95% confidence intervals) for baseline (full) and best-fitting (best) model of five leisure time activity scales for males and females.

Model	−2LL	Model fit statistics						Variance component estimates		
		df	Δ*χ* ^2^	Δdf	*P* value	AIC		*a* ^2^	*c* ^2^	*e* ^2^
Physical activity										
Full model	19074.67	2825	—	—	—	—	Males	0.15 (.00, .42)	0.36 (.13, .53)	0.48 (.41, .53)
							Females	0.49 (.23, .60)	0.05 (.00, .28)	0.46 (.40, .54)
Best model	19074.77	2827	0.74	2	0.69	−3.26	Males	0.07 (.00, .25)	0.43 (.27, .53)	0.50 (.43, .57)
							Females	0.54 (.47, .60)	—	0.46 (.40, .53)

Social										
Full model	17737.58	2826	—	—	—	—	Males	0.50 (.41, .56)	0.00 (.00, .00)	0.51 (.44, .59)
							Females	0.49 (.23, .61)	0.06 (.00, .28)	0.45 (.39, .53)
Best model	17739.71	2829	2.14	3	0.55	−3.86	Males	0.50 (.42, .57)	—	0.50 (.43, .58)
							Females	0.55 (.49, .61)	—	0.45 (.39, .51)

Intellectual										
Full model	17389.10	2826	—	—	—	—	Males	0.41 (.15, .50)	0.00 (.00, .00)	0.59 (.51, .68)
							Females	0.30 (.02, .47)	0.10 (.00, .32)	0.61 (.53, .71)
Best model	17390.91	2828	1.81	2	0.41	−2.20	Males	0.40 (.32, .49)	—	0.59 (.51, .68)
							Females	0.16 (.01, .40)	0.20 (.00, .35)	0.64 (.56, .73)

Family										
Full model	15966.60	2826	—	—	—	—	Males	0.31 (.00, .40)	0.00 (.00, .30)	0.69 (.60, .79)
							Females	0.23 (.00, .49)	0.20 (.00–.42)	0.57 (.50, .66)
Best model	15969.82	2829	3.22	3	0.36	−2.78	Males	0.31 (.21, .39)	—	0.69 (.61, .79)
							Females	0.44 (.36, .50)	—	0.56 (.50, .64)

Passive										
Full model	18186.62	2826	—	—	—	—	Males	0.03 (.00, .33)	0.21 (.00, .31)	0.76 (.66, .85)
							Females	0.30 (.07, .55)	0.23 (.01, .43)	0.46 (.40, .54)
Best model	18186.95	2828	0.33	2	0.85	−3.66	Males	—	0.23 (.15, .30)	0.77 (.70, .85)
							Females	0.35 (.15, .51)	0.19 (.05, .77)	0.46 (.40, .54)

Note: −2LL: −2 log-likelihood; df: degrees of freedom; AIC: Akaike information criterion; *a*: additive genetic; *c*: shared environment; *e*: nonshared environment.

**Table 6:** Parameter estimates (95% confidence intervals) for additive genetic (*a*), shared environment (*c*), and nonshared environmental (*e*) influences on five leisure time activities for males.

	Physical activity	Social activity	Intellectual activity	Family activity	Passive activity
*a*1	0.11 (.00, .33)				
*a*2	−0.10 (−.36, .24)	**0.39** **(.24, .49)**			
*a*3	0.06 (−.21, .40)	−0.39 (−1.31, .19)	**0.35** **(.18, .46)**		
*a*4	0.02 (−.24, .37)	−0.30 (−1.21, .37)	**0.39** **(.03, .77)**	**0.21** **(.02, .35)**	
*a*5	−1.69 (−1.69, 1.69)	0.54 (−.12, 1.04)	6.48 (.78, 6.48)	−0.27 (−1.89, .90)	**0.09** **(.02, .30)**
*c*1	**0.40** **(.20, .54)**				
*c*2	**0.52** **(.24, .75)**	**0.09** **(.03, .21)**			
*c*3	0.10 (−.22, .36)	**0.32** **(.01, .92)**	**0.06** **(.05, .20)**		
*c*4	0.33 (.02, .57)	0.05 (−.48, .52)	−0.16 (−.48, .11)	**0.09** **(.01, .26)**	
*c*5	5.01 (5.01, 5.01)	−0.06 (−.41, .42)	−0.35 (−.35, −.24)	0.00 (−1.15, 1.02)	**0.16** **(.01, .26)**
*e*1	**0.49** **(.42, .57)**				
*e*2	**0.58** **(.39, .78)**	**0.52** **(.44, .60)**			
*e*3	0.84 (.61, 1.12)	1.06 (.68, 1.68)	**0.59** **(.51, .68)**		
*e*4	**0.64** **(.45, .87)**	1.23 (.79, 1.01)	**0.79** **(.56, 1.04)**	1.28 (.68, 2.42)	
*e*5	−2.31 (−2.31, 2.31)	**0.52** **(.19, .91)**	−5.13 (−5.13, −.89)	1.28 (.67, 2.42)	**0.75** **(.73, .83)**

Note: bold indicates significant parameter estimates.

**Table 7:** Parameter estimates (95% confidence intervals) for additive genetic (*a*), shared environment (*c*), and nonshared environmental (*e*) influences on five leisure time activities for females.

	Physical activity	Social activity	Intellectual activity	Family activity	Passive activity
*a*1	**0.43** **(.25, .60)**				
*a*2	0.16 (−.29, .80)	**0.35** **(.31, .49)**			
*a*3	**0.45** **(.21, .64)**	0.71 (−1.33, 2.81)	**0.23** **(.06, .41)**		
*a*4	0.26 (−.15, .59)	1.01 (−.15, 2.51)	0.39 (−.12, .86)	**0.20** **(.02, .41)**	
*a*5	2.48 (2.48, 2.48)	0.45 (−.10, .97)	0.73 (−1.95, .73)	0.01 (−2.04, 1.64)	**0.30** **(.07, .52)**
*c*1	0.10 (.00, .25)				
*c*2	0.33 (−.19, .69)	**0.19** **(.07, .33)**			
*c*3	0.01 (−.11, .21)	−1.24 (−4.44, −.09)	**0.14** **(.01, .31)**		
*c*4	0.02 (−.23, .31)	−0.74 (−2.29, .21)	−0.03 (−.44, .40)	**0.22** **(.04, .42)**	
*c*5	−0.06 (−.06, −.06)	0.29 (−.17, .80)	1.55 (−.85, 1.55)	−0.23 (−.66, 1.79)	0.33 (−.19, .69)
*e*1	**0.47** **(.40, .54)**				
*e*2	**0.50** **(.30, .75)**	**0.46** **(.40, .53)**			
*e*3	**0.54** **(.39, .70)**	1.53 (.80, 4.40)	**0.63** **(.62, .71)**		
*e*4	0.73 (.47, 1.06)	0.72 (.30, 1.44)	**0.63** **(.43, .87)**	0.76 (.30, 1.69)	
*e*5	−1.42 (−1.42, 1.42)	0.26 (.06, .48)	−1.28 (−1.28, −.38)	0.76 (.28, 1.69)	**0.47** **(.41, .54)**

Note: bold indicates significant parameter estimates.

**Table 8:** Genetic, shared, and nonshared environmental correlations (95% confidence intervals) for five leisure time activities for males.

	1	2	3	4
*a*2	−0.13 (−.80, .22)			
*a*3	−0.08 (−.37, .42)	−0.15 (−.60, .08)		
*a*4	0.04 (−.68, .59)	−0.14 (−.67, .24)	**0.41** **(.04, 1.0)**	
*a*5	0.25 (−.50, .83)	−0.49 (−.10, .97)	−0.80 (−1.0, −.11)	−0.20 (−1.0, .51)
*c*2	0.77 (−.14, 1.0)			
*c*3	0.16 (−.52, .92)	0.66 (−.11, 1.0)		
*c*4	0.48 (.03, 1.0)	0.09 (−.55, .91)	−0.66 (−1.0, .50)	
*c*5	−0.30 (−.98, −.01)	−0.09 (−.74, .50)	0.08 (−.78, .96)	0.00 (−1.0, .72)
*e*2	**0.33** **(.23, .41)**			
*e*3	**0.39** **(.30, .47)**	**0.29** **(.20, .38)**		
*e*4	**0.30** **(.22, .39)**	**0.29** **(.19, .37)**	**0.34** **(.25, .43)**	
*e*5	0.06 (−.03, .15)	**0.15** **(.05, .24)**	**0.17** **(.07, .27)**	**0.19** **(.09, .28)**

Note: bold indicates significant parameter estimates.

**Table 9:** Genetic, shared, and nonshared environmental correlations (95% confidence intervals) for five leisure time activities for females.

	1	2	3	4
*a*2	0.10 (−.20, .44)			
*a*3	**0.51** **(.49, 1.0)**	0.20 (−.39, .69)		
*a*4	0.18 (−.13, .63)	0.44 (−.05, .1.0)	0.48 (−.11, .99)	
*a*5	−0.18 (−.50, .04)	0.30 (−.08, .71)	−0.18 (−.99, .33)	0.00 (−.92, .66)
*c*2	0.56 (−.77, .87)			
*c*3	0.04 (−.50, .48)	−0.61 (−.1.0, .09)		
*c*4	0.02 (−.41, .38)	−0.42 (−.90, .13)	−0.04 (−.75, .69)	
*c*5	0.01 (−.36, .37)	0.30 (−.18, .80)	−0.56 (−.62, −.22)	0.10 (−.72, .72)
*e*2	**0.24** **(.15, .34)**			
*e*3	**0.36** **(.27, .44)**	**0.23** **(.13, .32)**		
*e*4	**0.29** **(.20, .37)**	**0.16** **(.07, .25)**	**0.28** **(.19, .36)**	
*e*5	0.08 (−.02, .17)	**0.12** **(.03, .21)**	**0.16** **(.15, .24)**	**0.15** **(.06, .24)**

Note: bold indicates significant parameter estimates.
